# Fellowships, Grants, & Awards

**Published:** 2005-04

## Transportation Environmental Research Program (TERP)

Transportation and environmental issues are strongly connected, and in recent years legislation at all levels of government has further heightened the links between these issues. The Federal Highway Administration (FHWA) addresses these issues every day as complex policy decisions are made that balance mobility needs and environmental priorities. In order to understand these issues, the FHWA has engaged in many research efforts, but much of that research focuses on a multiregional or national level and can overlook the local effect. Consequently, the FHWA’s demand for research that is more focused and smaller in scale is growing. These types of research projects could provide a better understanding of the challenges faced by transportation practitioners.

The goal of the Transportation Environmental Research Program (TERP) is to add to the understanding of transportation and environmental issues and to continue the exploration of those issues through continued research and development work. The FHWA hopes that by committing to TERP, the research will provide insight on the difficult policy decisions that the FHWA will undertake in the future.

TERP grants of approximately $20,000 to $50,000 will be awarded based on the responses to TERP research announcements. The program is intended to be flexible, with the duration of each project ranging from six months to two years.

The research announcements are expected to fall into specific research topic areas, described as follows. Interested parties with related programs are encouraged to submit applications.

## Air Quality Conformity.

The Clean Air Act Amendments (CAAA), issued in 1990, mandate greater integration between transportation and air quality planning processes at all levels of government. The conformity regulations, issued in November 1993, set forth an elaborate mechanism to ensure that transportation planning is consistent with clean air objectives in nonattainment or maintenance areas for one or more of the following pollutants: ozone, carbon monoxide, particulate matter, and nitrogen dioxide. State and local agencies face a major challenge in meeting transportation-related CAAA provisions, including the conformity requirements. The FHWA is called upon to provide both technical and policy support to these agencies and other stakeholders. It is crucial that the FHWA has accurate, insightful, and up-to-the-minute information on 1) nationwide developments in air quality/transportation planning issues and 2) the strategies and practices of local and state agencies for compliance with federal transportation planning requirements.

## Air Quality Models.

The CAAA and complementary provisions of the Transportation Equity Act for the 21st Century strengthened controls on transportation to ensure that transportation activities contribute to the attainment of the National Ambient Air Quality Standards (NAAQS). As a result, a wide range of transportation projects have come under close scrutiny as potential contributors to air pollution.

Increases in travel demand have traditionally been satisfied through increased highway capacity. The conventional wisdom supporting this approach has been that increased capacity reduced congestion, fuel consumption, and emissions. This school of thought, however, has been challenged in recent years by analysts and planners concerned that the short-term benefits of smoother traffic flow may be degraded by increases in travel that are stimulated by increases in highway capacity. In addition, environmental groups frequently use legal challenges, allowed under the provisions of the CAAA, to limit increases in highway capacity.

## Emission Reduction (Transportation Strategies).

Emissions reduction efforts range from producing lower-emitting new vehicles to reducing vehicle use. The first efforts to reduce emissions focused on producing vehicles that emitted less pollution. In fact, extensive efforts have been made to control crankcase, evaporative, and exhaust emissions from new on-road vehicles through the implementation of emission standards for new vehicles.

Despite the progress in reducing the emission rates of new vehicles, emission system component failure, lack of proper maintenance, tampering, and the normal deterioration of emission control system performance all act to offset these emission benefits. In addition, most vehicles now in customer service were designed to comply with emissions standards for only five years or 50,000 miles. Overall, the effect of emission control system deterioration and vehicle longevity is that emission rates of a large portion of the vehicle fleet exceed those of new vehicles by as much as an order of magnitude or more.

Given the high degree to which emissions from new vehicles have been reduced, increased attention has been focused on control measures intended to reduce emissions from in-use vehicles. These reductions can come from technology improvements or technology-based control measures. Technology improvements offer a number of innovative approaches to reducing emissions. In fact, inspection and maintenance programs represent one of the first and perhaps most widespread of the technology-based control measures targeted at in-use vehicles. Other in-use technology-based control measures designed to reduce hydrocarbons and nitrogen oxide emissions that have been implemented in different areas of the United States include 1) gasoline volatility restrictions, 2) reformulated gasoline requirements, 3) vehicle scrappage programs, and 4) Stage II gasoline refueling vapor controls.

## NAAQS.

Technical and policy analysis of new ozone and particulate matter NAAQS revisions will be executed as deemed appropriate to assist FHWA staff in their efforts to 1) disseminate information on the scope of NAAQS revisions on the transportation planning process and 2) provide support to state and local transportation planning agencies.

## Mobile Source Air Toxics.

The impact of mobile source air toxics (MSATs) is emerging as one of the more challenging areas in the transportation–environmental community. Human health effects of MSATs is a growing concern, as the Environmental Protection Agency attempts to establish risk factors for many of the 21 identified toxics associated with transportation. Research into the methods of identifying, projecting, and mitigating MSAT impacts will be required.

## Environmental Impact Assessment in Transportation.

Although a significant amount of guidance exists, the approach to environmental impact assessment is often reinvented. Institutional and geographic impediments have hindered the definition of a standard or an accepted set of approaches to evaluating an issue, and the unique aspects of each project tend to blur the common elements. It will be the objective of this topic area to distill prototypical methods of impact assessment—through a hands-on case study approach that includes interviews and review of approved environmental documents; evaluation of commonly chosen methodologies, databases, and tools; and documentation of critical factors and barriers—and to provide for the transfer of successful impact assessment approaches to regional offices, National Environmental Policy Act (NEPA) practitioners, and municipal and state planning organizations.

The research under this topic area will be used to generate instructional materials with an immediate utility throughout the agency and in cooperative planning efforts between state and federal planning agencies. Care will be taken to highlight trends (areas where impact assessment is undergoing change) such as in wetland mitigation. In developing instructional materials it will be important to evaluate projects with a range of sizes and complexities, and to include as examples not only success stories but also projects perceived as failures. In addition to projects led by the FHWA, recipients should review other agencies—such as the U.S. Army Corps of Engineers—which also address issues critical to the environmental planning process.

## Environmental Justice, Housing Issues, and Environmental Laws.

No systematic database exists to show how far state departments of transportation (DOTs) have identified, and taken steps to mitigate, discriminatory effects of their projects, programs, and policies on low-income and minority populations. On 11 February 1994, President Clinton signed Executive Order 12898, “Federal Actions to Address Environmental Justice in Minority Populations and Low-Income Populations.” Two major federal laws, NEPA and Title VI of the Civil Rights Act, are highlighted in the order. Environmental justice and transportation equity concerns extend to discriminatory outcomes in planning, operation and maintenance, and infrastructure development by state and metropolitan systems. Discriminatory distributive transportation outcomes can be subsumed under three broad categories: procedural inequity, geographic inequity, and social inequity.

## Global Climate Change.

The composition of the Earth’s atmosphere is a primary determinant of the planet’s temperature, which in turn affects all life on Earth. Greenhouse gases occur naturally and trap heat within the atmosphere, helping to keep the planet hospitable to life. The main green-house gases are water vapor (H_2_O), carbon dioxide (CO_2_), methane (CH_4_), nitrous oxide (N_2_O), and halocarbons (such as chlorofluorocarbons, or CFCs). According to the U.S. Department of Energy, concentrations of greenhouse gases in the atmosphere have noticeably increased over the past 100 years.

Global climate change involves an increase in the average atmospheric temperature of the Earth. Such a temperature increase does not mean that temperatures will rise by a few degrees in all locations around the world. Rather, were global warming to occur, increases in atmospheric and oceanic temperatures might raise sea levels and alter associated weather patterns, which in turn could increase the frequency and severity of extreme weather worldwide. Such changes would likely alter current patterns of land use and human activity, as well as ecosystems and natural habitat.

## Right-of-Way Hazardous Substances, Materials, and Waste.

The first step in any approach to controlling highway-related hazardous substances is to inventory those materials most commonly related to highway projects and their rights-of-way (ROWs). This topic area will also evaluate the success and practicability of current methods and equipment used to restore or enhance existing sites located in ROWs. The key element that differentiates this topic area from the Hazardous Materials Generated During Development and Completion of Projects topic area is that the hazardous substances are identified early in the highway planning and acquisition process, thereby enabling the acquisition/environmental staff to conduct a thorough investigation to identify the extent of hazardous materials anticipated in the ROW.

## Hazardous Materials Generated During Development and Generation of Projects.

Hazardous materials are often exposed by earth-moving equipment as the ROW is cut to grade for subsequent construction. Occasionally, hazardous wastes are dumped at the construction site during off-duty hours, resulting in a need for rapid characterization and disposal of the dumped waste.

Typically, however, the soil contamination results from past disposal activities or past leakage of hazardous liquids into the soil—leakage that was undetected during the property acquisition phase. In either instance, the DOT will establish a rapid response investigative team, which is required to quickly characterize the waste, estimate the volume requiring remediation, and initiate an interim removal action. Prolonged delays in characterization and remediation of uncovered hazardous materials can lead to liquidated damages for the construction contractor.

In general, the options available to the DOT for remediation are limited to *ex situ* remedial actions including immediate excavation of the contaminated soil and transport to an alternative location for either treatment or disposal. This transport to another location belonging to the DOT, but removed from the ROW, enables construction to proceed while treatment of the contaminated soil is carried out.

## Stormwater Constituents.

Stormwater discharges from roads and highways represent an environmental issue requiring an understanding of not only the technical aspects of highway design and operations, site environmental impacts assessment, and regulatory requirements, but also the relative contribution and magnitude of the environmental impacts on the ecological system. Although available data show that highway stormwater discharges are most likely to have significant impact on localized areas, the holistic approach, which integrates highway stormwater runoff into the overall watershed-based ecological framework, allows for the evaluation of long-term water and water quality trends.

Monitoring of stormwater runoff from roads and highways continues to generate valuable information which, integrated with mathematical and statistical predictive models, can be used in various planning and engineering activities: 1) stormwater analysis and characterization of receiving water quality; 2) evaluation of ecological and human health impact analysis and compliance with water quality standards; 3) environmental impact assessment studies and compliance with associated regulatory requirements; 4) review, evaluation, and comparison of siting plans alternatives; 5) consideration of design alternatives to mitigate potentially significant impacts; 6) evaluation of stormwater management needs and development of pollution control programs; and 7) evaluation of water quality and ecological health of valuable and sensitive resources.

## Transportation Noise.

Noise, defined as unwanted or excessive sound, is an undesirable by-product of today’s society. It is often annoying, can interfere with sleep, work, or recreation, and in extremes may cause physical and psychological damage. Transportation noise is perhaps the most pervasive noise source and the most difficult to avoid in society today.

Noise impacts are not uniform with respect to vehicle miles traveled. One mile driven by a heavy truck on a local street creates much greater impact than a passenger car driven on an interstate with a landscaped shoulder. Given these differences, the level of highway traffic noise depends on three things: the volume of traffic, the speed of traffic, and the number of trucks in the flow of traffic. Noise associated with road transport is a combination of the noises produced from engine operations, exhaust, pavementγwheel contact, aerodynamic affects, and vibrating structures during operations. The loudness of traffic noise can also be increased by faulty equipment on vehicles, any condition that causes heavy laboring of motor vehicle engines (such as a steep incline), as well as more complicated factors (i.e., as a person moves away from a highway, traffic noise levels are reduced by distance, terrain, vegetation, and natural and manmade obstacles).

If noise impacts are identified, various noise abatement measures (for example, noise barriers) are considered to mitigate the adverse impacts. Other possible noise abatement measures include traffic management measures, creating buffer zones, planting vegetation, installing noise insulation in buildings, and relocating the highway.

## Water Quality.

Where possible, states, tribes, and other jurisdictions identify the pollutants that degrade water quality and indicators to document water quality degradation. Water quality monitoring is technically demanding and expensive. Furthermore, ideas about what indicators should be monitored and how to interpret the results continue to change.

Monitoring provides information that helps set policies and programs to protect and improve the quality of our nation’s streams, rivers, and lakes. It provides a basis for prioritizing needs so limited funds can be effectively allocated to improve conditions. Monitoring also provides the basis both for determining whether those policies and programs actually result in measurable environmental improvements, and for increasing the effectiveness of policies and programs.

## Ecosystem/Watershed Planning.

The watershed protection approach is a place-based strategy that integrates water quality management activities within hydrologically defined drainage basins, or watersheds, rather than areas defined by political boundaries. Thus, for a given watershed, the approach encompasses not only the water source (such as a stream, lake, estuary, or groundwater aquifer), but all the land from which water drains. To protect water resources, it is increasingly important to address the condition of land areas within the watershed because water carries the effects of human activities throughout the watershed as it drains off the land into surface waters or leaches into the groundwater.

Several key principles guide the watershed protection approach: place-based focus, stakeholder involvement and partnerships, environmental objectives, problem identification and prioritization, and integrated actions. The watershed protection approach is envisioned as the primary mechanism for achieving clean water and healthy, sustainable ecosystems throughout the nation. This approach enables stakeholders to take a comprehensive look at ecosystem issues and tailor corrective actions to meet local concerns within the coordinated framework of a national water program.

## Wetlands.

The proper application of functional evaluations is critical to mitigating adverse impacts of transportation projects on wetlands. Functional evaluations of newly created wetlands can be extremely useful in measuring the success of efforts to replace lost wetland functions. Such assessments require careful definition of objectives and a comparison of results to certain baseline conditions.

Depending on the objectives of the wetlands project, the baseline may be defined as the same wetland prior to alteration or as a nearby unaltered wetland of similar hydrogeomorphic type. Comparisons may also be made with other stated mitigation objectives, based on a reference wet-land representing a desired target condition.

Functional evaluations are also useful in providing performance standards for the design of new wetlands. The target condition for the proposed wetland can be defined based on the conditions of key factors related to capacity levels for the desired functions. With expert implementation of these approaches, newly created wetlands will be more successful in replacing impacted wet-land functions.

Although mitigation objectives are important, the primary considerations governing whether specific performance standards will be achieved are the site characteristics and their limitations. The objectives for replacing functions and functional capacity in the created wetland must be closely attuned to the site conditions, and this requires that the hydrologic, physical, chemical, and biological conditions at a mitigation site be characterized completely. The performance standards for the created wetland can then be based on those key functional capacity factors that correspond with conditions at the mitigation site.

After a proposed new wetland has been planned based on site limitations and opportunities, a functional evaluation may be conducted. The results of this evaluation can be compared with the stated mitigation objectives to determine whether a compensation ratio greater than 1:1 is necessary to replace functional capacity losses. When conducted properly, this process ensures that mitigation efforts will be sufficient to reach desired objectives and allows assessments of whether those objectives have been met in newly created wetlands.

All applications must be submitted on OMB Standard Forms 424, 424A, or 424B (as applicable), with the required information filled in and accompanied by the certifications required at 49 CFR Part 20, Appendix A and 49 CFR Part 29, Appendices A, B, and C. PDF versions of OMB Standard Forms 424, 424A, 424B, and the certification statements (items b through f) are available on the FHWA website at www.fhwa.dot.gov/aaa/forms2.htm. The complete online version of this announcement is available at http://www.fhwa.dot.gov/terp/prog.htm#I19.

Specific program announcements will be made in the Federal Business Opportunities web-site (http://www.eps.gov/) and on TERP’s website throughout the year (Section 3). The deadline for each submission will appear with each announcement and will occur approximately two months after the announcement is posted.

